# Microwave-assisted preparation and antimicrobial activity of *O*-alkylamino benzofurancarboxylates

**DOI:** 10.1007/s00706-013-1067-7

**Published:** 2013-08-16

**Authors:** Kinga Ostrowska, Elżbieta Hejchman, Irena Wolska, Hanna Kruszewska, Dorota Maciejewska

**Affiliations:** 1 Department of Organic Chemistry, Faculty of Pharmacy, Medical University of Warsaw, 1 Banacha, 02097 Warsaw, Poland; 2 Department of Crystallography, Faculty of Chemistry, Adam Mickiewicz University, Grunwaldzka 6, 60780 Poznan, Poland; 3 Department of Antibiotics and Microbiology, National Medicines Institute, 30/34 Chełmska, 00725 Warsaw, Poland

**Keywords:** Heterocycles, Alkylation, Phase-transfer catalysis, X-ray structure determination, Drug research

## Abstract

**Abstract:**

A series of derivatives of 2 and 3-benzofurancarboxylates were synthesized under microwave-assisted conditions. Their in-vitro antimicrobial properties were assessed. Inhibition by the compounds of the growth of antibiotic-susceptible standards and clinically isolated strains of Gram-positive and Gram-negative bacteria, yeasts, and a human fungal pathogen was moderate to significant. Methyl 5-bromo-7-[2-(*N*,*N*-diethylamino)ethoxy]-6-methoxy-2-benzofurancarboxylate hydrochloride was identified as the most active compound (MIC 3–12 × 10^−3^ μmol/cm^3^ against Gram-positive bacteria; MIC 9.4 × 10^−2^ μmol/cm^3^ against *Candida* and *Aspergillus brasiliensis*). The molecular and crystal structures of 2-(*N*,*N*-diethylamino)ethyl 6-acetyl-5-hydroxy-2-methyl-3-benzofurancarboxylate were established by single-crystal X-ray diffraction.

**Graphical Abstract:**

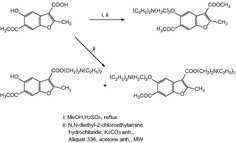
.

## Introduction

The benzofuran system, an important pharmacophore, is present in numerous compounds isolated from natural sources and in synthetic products. These heterocyclic compounds have a variety of pharmacological properties, and changes of their structure result in high diversity that has proved useful in the search for new therapeutic agents. It is widely known that numerous compounds containing the benzo[*b*]furan system, both synthetic and isolated from natural sources, have antimicrobial activity [1].

Eight flavaglines and six cyclopenta[*b*]benzofurans isolated from *Aglaia odorata*, *Aglaia elaeagnoidea*, and *Aglaia edulis* (Meliaceae) have been tested for antifungal properties against the three plant pathogens *Pyricularia grisea*, *Fusarium auenaceum*, and *Alternaria citri*. *P. grisea*, responsible for rice blast disease, was the fungus most susceptible to all the benzofurans, with rocaglaol the most active compound [2]. Thirteen compounds based on the benzofuran structure bearing aryl substituents at the C-3 position through a methanone linker have been synthesized and screened for antibacterial and antifungal activity against four bacteria: *Escherichia coli*, *Staphylococcus aureus*, Methicillin-resistant *S. aureus*, and *Bacillus subtilis*, and a fungus *Candida albicans*. Four hydrophobic benzofuran analogs were found to have favorable antibacterial activity better than that of control drugs [3].

It has been shown that esters and amides of 4-substituted 2-benzofurancarboxylic acids may act as inhibitors of fungal *N*-myristoyltransferase [4–8]. Mild to significant inhibition of the growth of an antibiotic-susceptible standard, clinically isolated strains of Gram-positive and Gram-negative bacteria, and human fungal pathogens was observed for a series of 2-substituted and three new diacetyl benzofurans. Different substitution of the benzofuran moiety and subsequent antimicrobial screening identified the C-3-acetyl functionality as a new structural alternative for optimum antimicrobial activity in the benzofuran class of compounds [9]. Substituted 3-methyl-2-benzofurancarbohydrazides had moderate activity against *S. aureus* and *B. subtilis* [10]. Similarly, 2-(1-benzofuran-2-yl-)-5-propyl-4,5-diphenyl-4,5-dihydrofuran-3-carbonitrile had average antimicrobial activity against *S. aureus*, *B. subtilis*, *Pseudomonas aeruginosa*, *Micrococcus luteus*, *E. coli*, *Salmonella enteritidis*, and *Listeria monocytogenes* [11]. Methyl esters of 4-bromo-6-(dibromoacetyl)-5-hydroxy-2-methyl-1-benzofuran-3-carboxylic acid (**I**), 6-(dibromoacetyl)-5-methoxy-2-methyl-1-benzofuran-3-carboxylic acid (**II**), and 4-chloro-6-(dichloroacetyl)-5-hydroxy-2-methyl-1-benzofuran-3-carboxylic acid (**III**) had antimicrobial activity against Gram-positive bacteria and compounds **I** and **III** had antifungal activity against *Candida albicans* and *C. parapsilosis* [12].

Surprisingly, no recently synthesized chloro and bromo derivatives of methyl 5-methoxy-2-methyl-3-benzofurancarboxylate had any antimicrobial activity [13].

As we have reported elsewhere, aminoalkylation the OH group of 7-hydroxycoumarin derivatives resulted in products with better antibacterial activity than the starting compounds [14]. Encouraged by this, and in continuation of our research, we designed the synthesis of a series of benzofurancarboxylates bearing *O*-aminoethyl substituents and assayed their antimicrobial activity. In this study we report their microwave-assisted preparation and discuss the advantages of this technique compared with synthesis under conventional conditions, described elsewhere [15]. The X-ray structure of 2-(*N*,*N*-diethylamino)ethyl 6-acetyl-5-hydroxy-2-methyl-3-benzofurancarboxylate (**1c**) is presented, with inter and intramolecular interactions in the solid state.

## Results and discussion

**Fig. 1:**
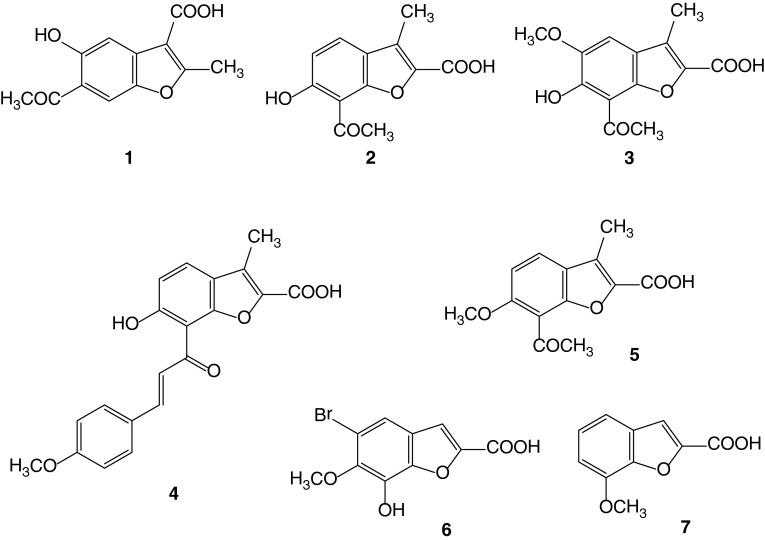
Structures of 2 and 3-benzofurancarboxylic acids

Our strategy was based on preparation of a series of derivatives of 2 and 3-benzofurancarboxylic acids (Fig. 1). Acids **1–6** were prepared as described elsewhere [15] and converted to their ammonium salts to improve solubility in polar solvents. Acids **1–4** and **6** were esterified with methanol to protect the carboxyl group against *O*-alkylation.

**Fig. 2:**
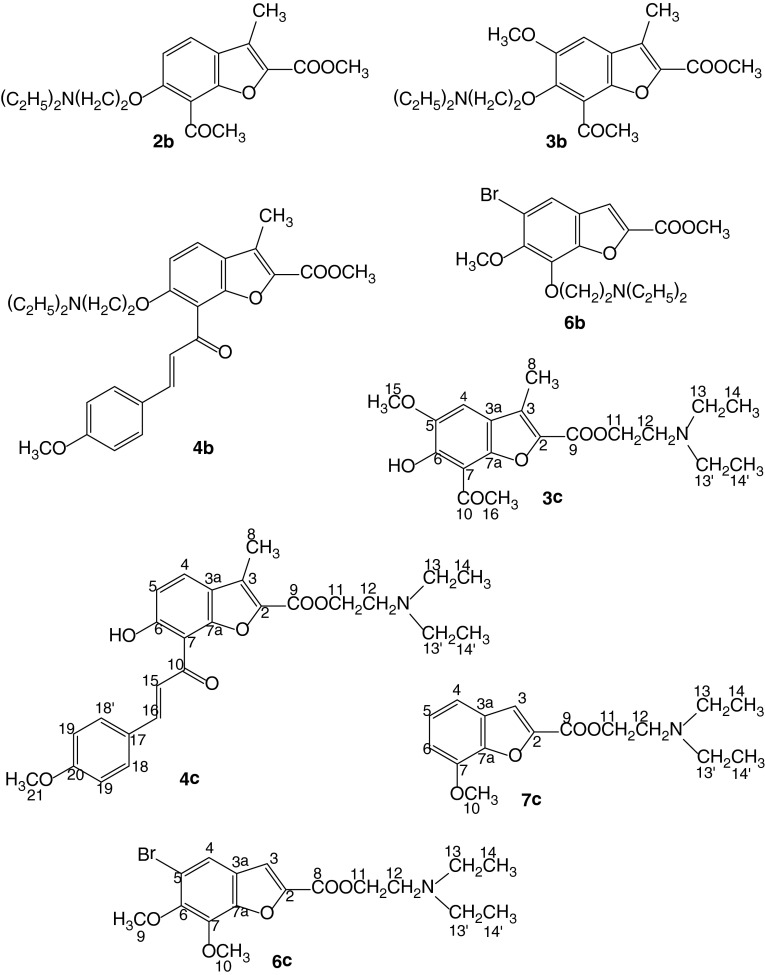
Structures of the esters of benzofurancarboxylic acids

As the first step of our research we obtained *O*-alkylamino derivatives of methyl benzofurancarboxylates **1b–4b** and **6b** by microwave-assisted *O*-alkylation of the appropriate esters (compounds **1a–4a**, **6a**, Scheme 1, routes i and ii, Fig. 2), using 2-chloroethyl-*N*,*N*-diethylamine hydrochloride as alkylating agent.

**Figure:**
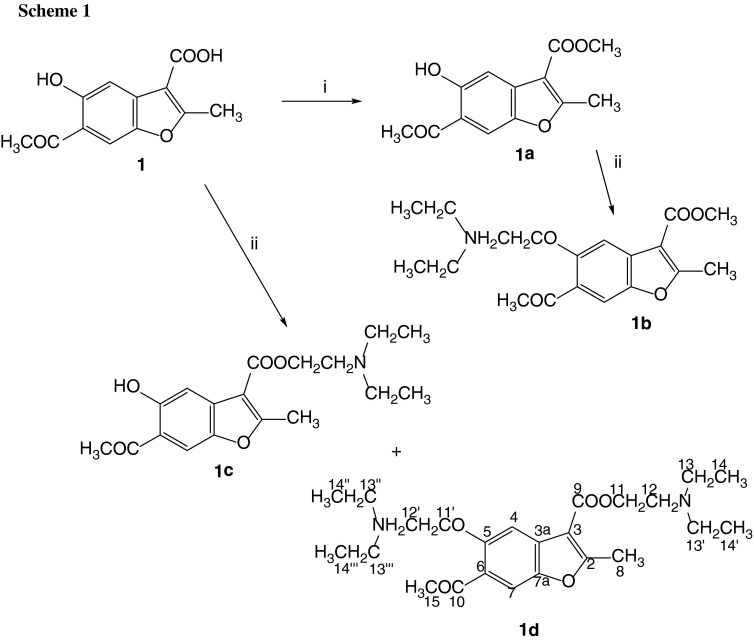

The syntheses were performed in acetone under phase-transfer conditions, using anhydrous potassium carbonate as a base and Aliquat 336 (*N*-methyl-*N*,*N*-dioctyloctan-1-ammonium chloride) as phase-transfer catalyst (PTC). Preparation of hydrochloride salts of the resulting bases was necessary to prevent decomposition and improve their solubility in polar solvents. These compounds were previously synthesized conventionally [15]. Microwave assistance resulted in reduced reaction time (from 16 to 20 h to 24 min); however, we did not notice any meaningful increase in product yield.

Benzofurancarboxylic acids **1–4**, **6**, and **7** reacted with 2-chloroethyl-*N*,*N*-diethylamine under similar conditions. Microwave-assisted alkylation of these compounds resulted in a mixture of two products. An example of this synthetic route (for compound **1**) is presented in Scheme 1, route ii. Separation by column chromatography on silica gel yielded the product of esterification **1c** and the product of *O*-alkylation and esterification **1d**. The isolated compounds **1c**, **1d**, **3c**, **4c**, **6c**, and **7c** (Fig. 2) were converted to their hydrochloride salts. Spectroscopic data (IR, ^1^H and ^13^C NMR, and MS) confirmed the structures of all the products.

In this investigation eighteen derivatives of 2 and 3-benzofurancarboxylic acids were assayed for in-vitro antimicrobial activity. The ammonium salts of benzofurancarboxylic acids **1–7** (Fig. 1) were also tested. They did not inhibit the growth of any of the microorganisms (MIC > 30 μmol/cm^3^). Methyl esters **1a–7a** of the acids [15] were not tested for antimicrobial activity.

Alkylation of hydroxyl groups in the molecules of methyl esters **1a–4a** and **6a** gave five 2-(*N*,*N*-diethylamino)ethoxy derivatives **1b–4b** and **6b** (Fig. 2; Scheme 1). All were evaluated microbiologically as hydrochloride salts. The in-vitro antimicrobial activity of compounds **1b**·HCl–**4b**·HCl and **6b**·HCl is summarized in Table 1.

**Table 1:** Antimicrobial activity of hydrochlorides of methyl benzofurancarboxylate *O*-alkylamino derivatives (minimum inhibitory concentration, μmol cm^−3^)

	**1b**·HCl	**2b**·HCl	**3b**·HCl	**4b**·HCl	**6b**·HCl
*Micrococcus luteus*	0.05	0.75	0.05	0.04	0.003
ATCC 9341					
*Bacillus cereus*	0.05	1.49	0.36	0.30	0.012
ATCC 11178					
*Bacillus subtilis*	0.05	1.49	0.18	0.04	0.012
ATCC 6633					
*Staphylococcus epidermidis*	0.05	1.49	0.18	0.04	0.012
ATCC 12228					
*Staphylococcus aureus*	0.10	3.11	0.18	0.15	0.012
ATCC 6538					
*Staphylococcus aureus*	0.05	3.11	0.18	0.15	0.012
ATCC 6538 P					
*Enterococcus hirae*	0.39	3.11	0.36	0.60	0.012
ATCC 10541					
*Escherichia coli*	6.51	12.44	6.04	NA	1.50
ATCC 8739					
*Pseudomonas aeruginosa*	13.02	NA	NA	NA	3.12
ATCC 15442					
*Candida albicans*	0.78	1.49	0.36	4.98	0.09
ATCC 10231					
*Candida albicans*	0.39	1.49	0.36	4.98	0.09
ATCC 2091					
*Candida parapsilosis*	0.39	1.49	0.72	0.2987	0.094
ATCC 22019					
*Saccharomyces cerevisiae*	NT	NT	NT	NT	0.187
ATCC 9763					
*Zygosacharomyces rouxi*	0.39	NT	0.36	NT	0.023
ATCC 28253					
*Aspergillus brasiliensis*	0.78	1.49	0.72	1.19	0.094
ATCC 16404					

*NA* not assayed >0.3 μmol/cm^3^, *NT* not tested

The results show that the pattern of substitution of the benzofuran moiety is important to the activity. The most potent compound is **6b**·HCl; at concentrations in the range 3–12 × 10^−3^ μmol/cm^3^ it inhibits growth of Gram-positive bacteria strains. Given its structure, we may speculate that the 2-(*N*,*N*-diethylamino)ethoxy function at C-7, the bromine substituent at C-5, and the methoxy group at C-6 are responsible for the high activity. The isomeric compound **6c**·HCl is, however, less active; exchanging the positions of the 2-(*N*,*N*-diethylamino)ethoxy and methoxy functions results in reduction of both antibacterial and antifungal activity.

It is worth noting that the derivative of the substituted 3-benzofurancarboxylic acid **1b**·HCl is more active against Gram-positive bacteria strains than compounds **2b**·HCl, **3b**·HCl, and **4b**·HCl, obtained from the substituted 2-benzofurancarboxylic acids. Introducing the lipophilic methoxy group at the C-5 position resulted in increased antimicrobial activity (compound **3b**·HCl is more active then **2b**·HCl). Similarly, the 7-(*p*-methoxycinnamoyl) group increases the activity of **4b**·HCl compared with **2b**·HCl against Gram-positive bacteria (Table 1). The 2-(*N*,*N*-diethylamino)ethyl esters **1c**·HCl, **3c**·HCl, and **4c**·HCl, with unsubstituted phenolic groups, are more active against Gram-positive bacteria but less active against Gram-negative bacteria than **1b**·HCl, **3b**·HCl, and **4b**·HCl (Table 2). It is worth noticing that compound **4c**·HCl is the most active against yeast strains. Compound **7c**·HCl was inactive in our assay.

**Table 2:** Antimicrobial activity of hydrochlorides of 2-(*N*,*N*-diethylamino)ethyl benzofurancarboxylates (minimum inhibitory concentration, μmol cm^−3^)

	**1c**·HCl	**3c**·HCl	**4c**·HCl	**6c**·HCl	**1d**·HCl	**7c**·HCl
*Micrococcus luteus*	0.01	0.01	0.01	0.09	0.04	15.28
ATCC 9341						
*Bacillus cereus*	0.10	0.05	0.04	0.71	0.04	15.28
ATCC 11778						
*Bacillus subtilis*	0.01	0.09	0.04	0.35	0.04	15.28
ATCC 6633						
*Staphylococcus epidermidis*	NA	0.19	0.01	0.09	0.04	15.28
ATCC 12228						
*Staphylococcus aureus*	1.62	0.19	0.01	0.09	0.04	15.28
ATCC 6538						
*Staphylococcus aureus*	0.41	0.09	0.04	0.18	0.07	15.28
ATCC 6538P						
*Enterococcus hirae*	NA	0.75	0.08	NA	0.30	**>**30.56
ATCC 10541						
*Escherichia coli*	NA	NA	NA	NA	0.59	**>**30.56
ATCC 8739						
*Pseudomonas aeruginosa*	NA	NA	NA	NA	NA	30.56
ATCC 15442						
*Candida albicans*	1.62	NA	0.32	5.91	2.47	**>**30.56
ATCC 10231						
*Candida albicans*	0.41	NA	0.08	NA	4.96	**>**30.56
ATCC 2091						
*Candida parapsilosis*	NA	NA	0.15	NA	4.96	**>**30.56
ATCC 22019						
*Saccharomyces cerevisiae*	1.62	3.13	0.08	1.42	4.96	15.28
ATCC 9763						
*Zygosacharomyces rouxi*	NA	NA	0.04	5.91	0.59	**>**30.56
ATCC 28253						
*Aspergillus brasiliensis*	NA	NA	NA	NA	4.96	15.28
ATCC 16404						

*NA* not assayed >0.3 μmol/cm^3^; *NT* not tested

### X-ray structure analysis

**Fig. 3:**
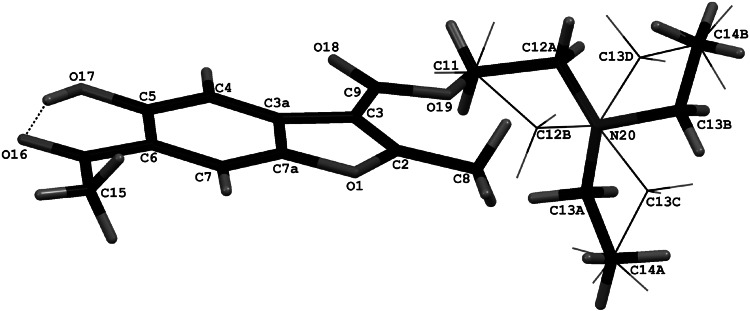
Schematic diagram of molecule **1c** showing the labeling scheme and the disordered 2-(*N*,*N*-diethylamino)ethyl substituent

The molecular and crystal structure of **1c** in the solid state were analyzed by single-crystal X-ray diffraction. The molecular structure with the atomic numbering scheme is illustrated in Fig. 3 (the drawings were performed with Mercury software [16]). The results indicate that the compound crystallizes in the monoclinic space group *P* 2_1_/*n* with one molecule in the asymmetric unit. Selected bond lengths, bond angles, and torsion angles are listed in Table 3. The benzofuran moiety is nearly planar with a maximum deviation of 0.020(1) Å for C3a. The C8, C9, C10, O16, O17, and O18 atoms are almost coplanar with the two-ring framework (the appropriate torsion angles are given in Table 3). The orientation of the substituent at C3 relative to the benzofuran ring can be described by the torsion angle C2–C3–C9–O19 of −0.2(3)°. For the (*N*,*N*-diethylamino)ethyl fragment we observed structural disorder as a result of conformational freedom and from X-ray data we found alternative positions of the C12 and C13 atoms. Strong intramolecular hydrogen bonding is present between O16 and O17 atoms (Fig. 3; Table 4). The angle between the best planes of the benzofuran moiety and the C5/O17/H17A/O16/C10/C6 ring is only 1.56(6)°. Moreover the weak C4–H4A···O18 and C11–H11···O18 interactions stabilize the conformation of the molecule.

**Table 3:** Selected bond lengths/Å and angles/°, and selected torsional angles/° for **1c**

O1–C2	1.309(2)
O1–C7a	1.384(2)
C5–O17	1.351(2)
C3a–C7a	1.331(2)
C6–C10	1.433(3)
C2–C8	1.477(2)
C2–O1–C7a	108.6(1)
C3–C9–O19	112.5(1)
C6–C10–O16	123.2(2)
C3a–C3–C2–C8	177.1(2)
C7–C6–C5–O17	179.0(2)
C2–C3–C9–O18	178.8(2)
O1–C2–C3–C9	−178.3(2)
C7–C6–C10–O16	−178.1(2)
C4–C5–C6–C10	179.6(2)

**Table 4:** Intra and intermolecular interactions in crystals of **1c** (Å, °)

D-H···A	D-H	H···A	D···A	<(D-H···A)
O17–H17A···O16	0.82	1.70	2.433(2)	148
C4–H4A···O18	0.93	2.54	3.011(2)	111
C11–H11D···O18	0.97	2.28	2.637(3)	101
C7–H7A···O18^a^	0.93	2.53	3.313(2)	179
C11–H11A···O1^b^	0.97	2.53	3.166(2)	123
C13D–H13G···O17^c^	0.97	2.71	3.415(3)	130
C13C–H13F···O16^d^	0.97	2.67	3.435(5)	136
C8–H8B···C10^e^	0.96	2.85	3.661(3)	143
C15–H15C···C9^f^	0.96	2.84	3.558(3)	133

Symmetry codes: ^a^1 + x, y, z; ^b^−1 + x, y, z; ^c^−x, 1 − y, −z; ^d^−0.5 + x, 1.5 − y, 0.5 + z; ^e^1 − x, 1 − y,−z; ^f^1 − x, 2 − y, −z

**Fig. 4:**
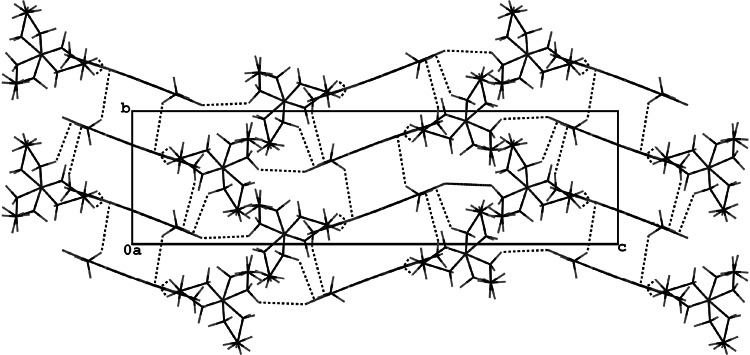
Projection of the crystal structure of **1c** viewed along the *a* axis, showing molecular blocks

The packing of the molecules viewed down the *a* axis (Fig. 4) shows that the molecules are stacked in blocks with partly overlapping benzofuran systems and an interlayer spacing of ca. 3.5 Å. The molecules are linked by C7–H7A···O18, C11–H11A(D)···O1 hydrogen bonds forming infinite chains along the *a* axis. These chains interact via C13D–H13G···O17, C15–H15C···C9, C8–H8B···C10 contacts and *π*···*π* stacking forces to create the blocks mentioned above. The bulky aminoethyl substituents are oriented outside these blocks and connect them via C13C–H13F···O16 hydrogen bonds. Geometric data for all intra and intermolecular interactions are given in Table 4.

## Experimental

Reagents of the highest grade available were purchased from Aldrich and used without further purification. Solvents were used as received from commercial suppliers, and no further attempts were made to purify or dry them. Melting points were determined with an ElectroThermal 9001 digital melting point apparatus (ElectroThermal, Essex, UK). A Plazmatronika 1,000-W microwave oven equipped with a single mode cavity suitable for microscale synthesis and microwave choked outlet connected to an external condenser set to 30 % power was used (http://www.plazmatronika.com.pl). High-resolution mass spectra were recorded on a Quattro LCT (TOF). ^1^H NMR, ^13^C NMR, HSQC, and HMBC spectra in solution were recorded at 25 °C with Varian NMRS-300 or a Varian Unity plus-500 spectrometers, and standard Varian software was used (Varian, Palo Alto, CA, USA). Calculated shielding constants were used as an aid to assignment of resonances of ^13^C atoms. The CPHF-GIAO approach was used for computation of NMR shielding constants using Gaussian 09 software [17]. Chemical shifts (*δ*, ppm) were referenced to TMS. The notation used for detailed description of NMR resonances is given in Scheme 1 and Fig. 2. IR spectra were recorded on a Perkin Elmer FT IR Spectrum 2000 instrument. TLC was performed on silica gel 60 F_254_ sheets (Merck, Darmstadt, Germany), spots were visualized by UV at 254 and 365 nm. Silica gel 60 was used for column chromatography. Preparation of compounds **1b**–**6b** has been described elsewhere [15].

### General procedure for microwave-assisted preparation of hydrochlorides of methyl [2-(*N,N*-diethylamino)ethoxy]-substituted benzofurancarboxylates

A mixture of the appropriate methyl benzofurancarboxylate (2 mmol), *N*,*N*-diethyl-2-chloroethylamine hydrochloride (6 mmol), anhydrous potassium carbonate (23 mmol), and Aliquat 336 (0.25 mmol) in 10 cm^3^ anhydrous acetone was placed in the microwave flask and heated under reflux in the monomode microwave oven for 24 min. The reaction was monitored by TLC. After completion of the reaction inorganic salts were removed by filtration. The solvent was evaporated. The residue was purified by column chromatography on silica gel, eluent: CHCl_3_–MeOH 50:1. The base was dissolved in methanol saturated with gaseous HCl. The hydrochloride was precipitated by addition of diethyl ether. The crude product was crystallized from methanol–diethyl ether.

#### Methyl 6-acetyl-5-[2-(*N,N*-diethylamino)ethoxy]-2-methyl-3-benzofurancarboxylate (**1b**, C_19_H_25_NO_5_)

^1^H NMR (300 MHz, CDCl_3_): *δ* = 1.11 (t, *J* = 7.2 Hz, 6H, H-14,14′), 2.69 (s, 3H, H-16), 2.70 (m, 4H, H-13,13′), 2.77 (s, 3H, H-8), 2.99 (t, *J* = 6.3 Hz, 2H, H-12), 3.96 (s, 3H, H-15), 4.25 (t, *J* = 6.3 Hz, 2H, H-11), 7.49 (s, 1H, H-4), 7.82 (s, 1H, H-7) ppm; ^13^C NMR (125 MHz, CDCl_3_): *δ* = 11.66 (C-14,14′), 15.10 (C-8), 32.16 (C-16), 47.92 (C-13), 51.81 (C-15), 52.02 (C-12), 67.45 (C-11), 104.70 (C-4), 109.25 (C-3), 112.51 (C-7), 125.88 (C-6), 131.05 (C-3a), 148.20 (C-7a), 155.68 (C-5), 164.62 (C-9), 167.40 (C-2), 199.34 (C-10) ppm.

#### **1b**·HCl (C_19_H_26_ClNO_5_)

^1^H NMR (300 MHz, CDCl_3_): *δ* = 1.48 (t, *J* = 6.9 Hz, 6H, H-14,14′), 2.62 (s, 3H, H-16), 2.71 (m, 4H, H-13,13′), 2.79 (br.s, 3H, H-8), 3.21 (m, 4H, H-13, 13′), 3.55 (m, 2H, H-12), 3.98 (s, 3H, H-15), 4.71 (br.s, 2H, H-11), 7.55 (s, 1H, H-4), 7.74 (s, 1H, H-7), 12.47 (br.s, 1H, NH) ppm.

#### Methyl 7-acetyl-6-[2-(*N,N*-diethylamino)ethoxy]-3-methyl-2-benzofurancarboxylate hydrochloride (**2b**·HCl, C_19_H_26_ClNO_5_*x*H_2_O), methyl 7-acetyl-6-[2-(*N,N*-diethylamino)ethoxy]-5-methoxy-3-methyl-2-benzofurancarboxylate hydrochloride (**3b**·HCl, C_20_H_28_ClNO_6_), methyl 6-[2-(*N,N*-diethylamino)ethoxy]-7-(p-methoxycinnamoyl)-3-methyl-2-benzofurancarboxylate hydrochloride (**4b**·HCl, C_27_H_32_ClNO_6_), methyl 5-bromo-7-[2-(*N,N*-diethylamino)ethoxy]-6-methoxy-2-benzofurancarboxylate hydrochloride (**6b**·HCl, C_17_H_22_BrNO_5_)

Analytical data (^1^H NMR data and m.p.) for compounds **2b**–**4b** and **6b** were in agreement with the data reported in our paper [15].

### General procedure for microwave-assisted preparation of hydrochlorides of 2-(*N,N*-diethylamino)ethyl benzofurancarboxylates

The appropriate benzofurancarboxylic acid (0.3 mmol), *N*,*N*-diethyl-2-chloroethylamine hydrochloride (1.5 mmol), anhydrous potassium carbonate (10.2 mmol), and Aliquat 336 (0.25 mmol) in 8 cm^3^ anhydrous acetone were placed in the microwave flask. The mixture was heated under reflux in the monomode microwave oven: 4–8 cycles: heating 6 min, cooling 2 min. TLC monitoring on silica gel plates (mobile phase CHCl_3_–MeOH 10:1) indicated complete disappearance of the substrate. The inorganic salts were removed by filtration, then the solvent was evaporated. The residue was purified by column chromatography on silica gel 230–400 mesh, eluent: CHCl_3_–MeOH 50:1. One or two basic products were isolated. The bases were converted into their hydrochlorides as described above.

#### 2-(*N,N*-Diethylamino)ethyl 6-acetyl-5-hydroxy-2-methyl-3-benzofurancarboxylate (**1c**, C_18_H_23_NO_5_)

Yield 61 %; m.p.: 101–103 °C; *R*_f_ = 0.69; ^1^H NMR (300 MHz, CDCl_3_): *δ* = 1.10 (t, 6H, *J* = 7.2 Hz, H-14,14′), 2.67 (t, 4H, *J* = 7.2 Hz, H-13,13′), 2.68 (s, 3H, H-15), 2.79 (s, 3H, H-8), 2.91 (t, 2H, *J* = 6.5 Hz, H-12), 4.45 (t, 2H, *J* = 6.5 Hz, H-11), 7.49 (s, 1H, H-4), 7.77 (s, 1H, H-7), 12.17 (1H, OH) ppm; ^13^C NMR (125 MHz, CDCl_3_): *δ* = 11.74 (C-14,14′), 15.13 (C-8), 26.94 (C-16), 47.82 (C-13,13′), 51.36 (C-12), 62.28 (C-11), 109.37 (C-3), 109.46 (C-4), 111.97 (C-7), 116.45 (C-6), 134.08 (C-3a), 146.77 (C-7a), 159.45 (C-5), 163.83 (C-9), 169.56 (C-2), 203.88 (C-10) ppm; IR (CHCl_3_): $\bar{\nu}$ = 3,417 ($\nu_{OH}$), 3,076 ($\nu_{{{\text{C}} - {\text{H}}_{\text{arom}} }}$), 2,963, 2,926 ($\nu_{{{\text{C}} - {\text{H}}_{\text{asym}} }}$), 2,852 ($\nu_{{{\text{C}} - {\text{H}}_{\text{sym}} }}$), 1,703 ($\nu_{{{\text{C}} = {\text{O}}}}$), 1,621, 1,587 ($\nu_{{{\text{C}} = {\text{C}}}}$), 1,423 (*δ*_OH_), 1,318, 1,260 ($\nu_{{{\text{C}}--{\text{O}}--{\text{C}}_{\text{asym}} }}$), 1,176, 1,092 ($\nu_{{{\text{C}}--{\text{O}}--{\text{C}}_{\text{asym}} }}$), 979, 887, 863, 799 ($\gamma_{{{\text{C}} - {\text{H}}}}$) cm^−1^; MS (TOF-ES+): [M + H]^+^ calcd for C_18_H_24_NO_5_ 334.1654, found 334.1654.

#### **1c**·HCl (C_18_H_24_ClNO_5_)

^1^H NMR (300 MHz, CDCl_3_): *δ* = 1.46 (t, 6H, *J* = 7.2 Hz, H-14,14′), 2.79 (s, 3H, H-8), 2.69 (s, 3H, H-16), 3.25 (m, 4H, H-13,13′), 3.46 (m, 2H, H-12), 4.94 (t, 2H, *J* = 5.4 Hz, H-11), 7.35 (s, 1H, H-4), 7.80 (s, 1H, H-7) ppm, 12.17 (1H, OH) ppm; ^13^C NMR (125 MHz, CDCl_3_): *δ* = 8.87 (C-14,14′), 15.41 (C-8), 26.98 (C-16), 47.52 (C-13,13′), 50.13 (C-12), 58.57 (C-11), 108.37 (C-3), 108.90 (C-4), 112.33 (C-7), 116.70 (C-6), 133.43 (C-3a), 146.75 (C-7a), 159.61 (C-5), 163.21 (C-9), 170.51 (C-2), 203.86 (C-10) ppm.

#### 2-(*N,N*-Diethylamino)ethyl 6-acetyl-5-[2-(*N,N*-diethylamino)ethoxy]-2-methyl-3-benzofurancarboxylate (**1d**, C_24_H_36_N_2_O_5_)

Yield 71 %; m.p.: 101–103 °C; *R*_f_ = 0.75; ^1^H NMR (300 MHz, CDCl_3_): *δ* = 1.068, 1.072 (t, 6H, *J* = 7.2 Hz; t, 6H, *J* = 7.8 Hz; H-14,14′,14″,14′′′), 2.64 (q, 8H, *J* = 7.2 Hz, H-13, 13′, 13″,13′′′), 2.70 (s, 3H, H-15), 2.77 (s, 3H, H-8), 2.87 (t, 2H, *J* = 6.3 Hz, H-12′), 2.95 (t, 2H, *J* = 6.3 Hz, H-12), 4.20 (t, 2H, *J* = 6.3 Hz, H-11′), 4.44 (t, 2H, *J* = 6.3 Hz, H-11), 7.54 (s, 1H, H-4), 7.83 (s,1H, H-7) ppm; MS (TOF-ES+): [M + H]^+^ calcd for C_24_H_37_N_2_O_5_ 433.2702, found 433.2702.

#### **1d**·2HCl (C_24_H_38_Cl_2_N_2_O_5_)

^1^H NMR (300 MHz, CDCl_3_): *δ* = 1.44 (t, 6H, *J* = 7.2 Hz), 1.47 (t, 6H, *J* = 7.8 Hz, H-14,14′), 2.63 (s, 3H, H-16), 2.79 (s, 3H, H-8), 3.35 (m, 8H, H-13, 13′, 13″,13′′′), 3.61 (t, 2H, *J* = 5.1 Hz, H-12′), 3.72 (m, 2H, H-12), 4.86 (t, *J* = 4.8 Hz, 2H, H-11′), 4.98 (t, *J* = 4.8 Hz, 2H, H-11), 7.69 (s, 1H, H-4), 7.76 (s, 1H, H-7), 11.92, 12.04 (br.s, 2 NH) ppm; IR (CHCl_3_): $\bar{\nu}$ = 2,981, 2,955, 2,927 ($\nu_{{{\text{C}}--{\text{H}}_{\text{asym}} }}$), 2,855 ($\nu_{{{\text{C}}--{\text{H}}_{\text{asym}} }}$), 2,489 ($\nu_{{{\text{N}}--{\text{H}}}}$ tertiary amine salt), 1,713 ($\nu_{{{\text{C}} = {\text{O}}}}$), 1,623, 1,588 ($\nu_{{{\text{C}} = {\text{C}}}}$), 1,440 ($\delta_{{{\text{C}}--{\text{H}}_{\text{asym}} }}$), 1,250, 1,227 ($\nu_{{{\text{C}}--{\text{O}} - {\text{C}}_{\text{asym}} }}$), 1,182, 1,090 ($\nu_{{{\text{C}} - {\text{O}} - {\text{C}}_{\text{sym}} }}$), 979, 892, 847, 780 ($\gamma_{{{\text{C}}--{\text{H}}}}$) cm^−1^.

#### 2-(*N,N*-Diethylamino)ethyl 7-acetyl-6-hydroxy-5-methoxy-3-methyl-2-benzofurancarboxylate (**3c**, C_19_H_25_NO_6_)

Yield 55 %; m.p.: 61–63 °C; *R*_f_ = 0.16; ^1^H NMR (300 MHz, CDCl_3_): *δ* = 1.12 (t, 6H, *J* = 7.2 Hz, H-14,14′), 2.57 (s, 3H, 8-H), 2.72 (q, 4H, *J* = 7.2 Hz, H-13,13′), 2.95 (s, 3H, H-16), 2.94 (t, 2H, *J* = 6.0 Hz, H-12), 3.98 (s, 3H, H-15), 4.93 (t, 2H, *J* = 6.0 Hz, H-11), 7.15 (s, 1H, H-4), 13.61 (br.s, 1H, OH) ppm; ^13^C NMR (125 MHz, CDCl_3_): *δ* = 9.55 (C-8), 11.72 (C-14,14′), 31.76 (C-16), 47.85 (C-13,13′), 51.19 (C-12), 56.85 (C-15), 62.71 (C-11), 106.93 (C-7), 107.64 (C-4), 119.74 (C-3a), 126.80 (C-3), 140.32 (C-2), 146.97 (C-6), 147.99 (C-5), 156.37 (C-7a), 160.00 (C-9), 203 (C-10) ppm; MS (TOF-ES+): [M + H]^+^ calcd for C_19_H_26_NO_6_ 362.1760, found 364.1760.

#### **3c**·HCl (C_19_H_26_ClNO_6_)

IR (CHCl_3_): 3,424 ($\nu_{\text{OH}}$), 2,954, 2,926 ($\nu_{{{\text{C}} - {\text{H}}_{\text{asym}} }}$), 2,855 ($\nu_{{{\text{C}} - {\text{H}}_{\text{asym}} }}$), 2,485 ($\nu_{{{\text{N}}--{\text{H}}}}$ tertiary amine salt), 1,716 ($\nu_{{{\text{C}} = {\text{O}}}}$), 1,610, 1,584 ($\nu_{{{\text{C}} = {\text{C}}}}$), 1,428 (*δ*_OH_), 1,316, 1,260 (${{\upnu}}_{{{\text{C}}--{\text{O}} - {\text{C}}_{\text{asym}} }}$), 1,148, 1,097 (${{\upnu}}_{{{\text{C}}--{\text{O}} - {\text{C}}_{\text{asym}} }}$), 977, 934, 848, 799, 769 ($\gamma_{{{\text{C}}--{\text{H}}}}$) cm^−1^.

#### 2-(*N,N*-Diethylamino)ethyl 6-hydroxy-7-(p-methoxycinnamoyl)-3-methyl-2-benzofurancarboxylate (**4c**, C_26_H_29_NO_6_)

Yield 65 %; m.p.: 104–106 °C; *R*_f_ = 0.59; ^1^H NMR (300 MHz, CDCl_3_): *δ* = 1.08 (t, 6H, *J* = 7.2 Hz, H-14,14′), 2.57 (s, 3H, H-8), 2.67 (m, 4H, H-13,13′), 2.96 (t, 2H, *J* = 6.5 Hz, H-12), 3.88 (s, 3H, H-21), 4.53 (t, 2H, *J* = 6.5 Hz, H-11), 6.97 (m, 4H, H-18,18′,19,19′), 7.65 (d, 1H, *J* = 8.7 Hz, H-5), 7.75 (d, 1H, *J* = 8.7 Hz, H-4), 7.99 (d, 1H, *J* = 15.3 Hz, H-16), 8.34 (d, 1H, *J* = 15.1 Hz, H-15), 14.06 (br.s, 1H, OH) ppm; ^13^C NMR (125 MHz, CDCl_3_): *δ* = 9.40 (C-14,14′), 11.96 (C-8), 29.91 (C-16), 47.81 (C-13,13′), 51.51 (C-12), 55.69 (C-21), 62.86 (C-11), 107.35 (C-3), 114.79 (C-19,19′), 116.03 (C-5), 121.64 (C-7), 122.69 (C-4), 127.92 (C-17), 128.35 (C-3a), 131.11 (C-18, 18′), 140.19 (C-7a), 145.94 (C-15), 153.59 (C-20), 160.01 (C-6), 162.36 (C-9), 166.54 (C-2), 191.85 (C-10) ppm; IR (CHCl_3_): $\bar{\nu}$ = 3,400 ($\nu_{\text{OH}}$), 2,965, 2,925 ($\nu_{{{\text{C}} - {\text{H}}_{\text{asym}} }}$), 2,851 ($\nu_{{{\text{C}} - {\text{H}}_{\text{asym}} }}$), 1,712 ($\nu_{{{\text{C}} = {\text{O}}}}$), 1,635, 1,593 ($\nu_{{{\text{C}} = {\text{C}}}}$), 1,424 (*δ*_OH_), 1,259 ($\nu_{{{\text{C}}--{\text{O}} - {\text{C}}_{\text{asym}} }}$), 1,172, 1,085 ($\nu_{{{\text{C}}--{\text{O}} - {\text{C}}_{\text{asym}} }}$), 983, 869, 829, 764 ($\gamma_{{{\text{C}}--{\text{H}}}}$) cm^**−**1^; MS (TOF-ES+): [M + H]^+^ calcd for C_26_H_30_NO_6_ 452.3222, found 452.3222.

#### 2-(*N,N*-Diethylamino)ethyl 5-bromo-6,7-dimethoxy-2-benzofurancarboxylate (**6c**, C_17_H_22_BrNO_5_)

This compound was prepared in accordance with the general procedure, except acetone–methanol 9:10 was used instead of anhydrous acetone. Yield 48 %; m.p.: 61–63 °C; *R*_f_ = 0.52; ^1^H NMR (300 MHz, CDCl_3_): *δ* = 1.11 (t, 6H, *J* = 7.2 Hz, H-14,14′), 2.75 (q, 4H, *J* = 7.2 Hz, H-13,13′), 3.00 (t, 2H, *J* = 6.3 Hz, H-12), 3.92 (s, 3H, H-10), 3.95 (s, 3H, H-9), 4.44 (t, 2H, *J* = 6.3 Hz, H-11), 7.12 (s, 1H, 3-H), 7.46 (s, 1H, 4-H) ppm; MS (TOF-ES+): [M + H]^+^ calcd for C_17_H_23_NO_5_Br^79^ 400.0753, found 400.1958; C_17_H_23_NO_5_Br^81^ 402.0733, found 402.2709.

#### **6c**·HCl **(**C_17_H_23_BrClNO_5_)

^1^H NMR (300 MHz, CDCl_3_): *δ* = 1.14 (t, 6H, *J* = 7.5 Hz, H-14,14′), 2.82 (m, 2H, H-13,13′), 3.05 (m, 2H, H-12), 3.92 (s, 3H, H-10), 3.95 (s, 3H, H-9), 4.62 (t, 2H, *J* = 6.3 Hz, H-11), 7.13 (s, 1H, 3-H), 7.46 (s, 1H, 4-H) ppm; IR (CHCl_3_): $\bar{\nu }$ = 2,967, 2,932 ($\nu_{{{\text{C}} - {\text{H}}_{\text{asym}} }}$), 2,848 ($\nu_{{{\text{C}} - {\text{H}}_{\text{asym}} }}$), 1,731 ($\nu_{{{\text{C}} = {\text{O}}}}$), 1,619, 1,581, 1,502 ($\nu_{{{\text{C}} = {\text{C}}}}$), 1,262 ($\nu_{{{\text{C}}--{\text{O}} - {\text{C}}_{\text{asym}} }}$), 1,122 ($\nu_{{{\text{C}}--{\text{O}} - {\text{C}}_{\text{asym}} }}$), 982, 914, 834, 764 ($\gamma_{{{\text{C}}--{\text{H}}}}$) cm^−1^.

#### 2-(*N,N*-Diethylamino)ethyl 7-methoxy-2-benzofurancarboxylate (**7c**, C_16_H_21_NO_4_)

Yield 55 %; oil; *R*_f_ = 0.86; ^1^H NMR (300 MHz, CDCl_3_): *δ* = 1.09 (t, 6H, *J* = 7.2 Hz, H-14,14′), 2.66 (q, 4H, *J* = 6.9 Hz, H-13,13′), 2.89 (t, 2H, *J* = 6.3 Hz, H-12), 4.02 (s, 3H, H-10), 4.58 (t, 2H, *J* = 6.3 Hz, H-11), 6.92 (dd, 1H, *J* = 7.2 Hz, 1.5 Hz, H-5), 7.24 (m, 2H, H-4, H-6), 7.51 (s, 1H, H-3) ppm; MS (TOF-ES+): [M + H]^+^ calcd for C_16_H_22_NO_4_ 292.1555, found 292.1549.

#### **7c**·HCl (C_16_H_22_ClNO_4_)

IR (CHCl_3_): $\bar{\nu }$ = 2,979, 2,950 ($\nu_{{{\text{C}} - {\text{H}}_{\text{asym}} }}$), 2,843 ($\nu_{{{\text{C}} - {\text{H}}_{\text{asym}} }}$), 2,485 ($\nu_{{{\text{N}}--{\text{H}}}}$ tertiary amine salt), 1,727 ($\nu_{{{\text{C}} = {\text{O}}}}$), 1,622, 1,594 ($\nu_{{{\text{C}} = {\text{C}}}}$), 1,366, 1,307, 1,271 ($\nu_{{{\text{C}}--{\text{O}}--{\text{C}}_{\text{asym}} }}$), 1,185, 1,093 ($\nu_{{{\text{C}}--{\text{O}}--{\text{C}}_{\text{asym}} }}$), 972, 914, 850, 800, 780, 732 ($\gamma_{{{\text{C}}--{\text{H}}}}$) cm^−1^.

### Microbiology

The following microbial strains with different cell wall structures were chosen:

- Gram-positive bacteria: *Micrococcus luteus* ATCC 9341, *B. cereus* ATCC 11778, *B. subtilis* ATCC 6633, *S. epidermidis* ATCC 12228, *S. aureus* ATCC 6538, *S. aureus* ATCC 6538P, *E. hirae* ATCC 10541;
- Gram-negative bacteria: *E. coli* ATCC 8739, *P. aeruginosa* ATCC 15442; and
- fungal strains: *Aspergillus brasiliensis* ATCC 16404, *C. albicans* ATCC 10231 and ATCC 2091, *C. parapsilosis* ATCC 22019, *S. cervisiae* ATCC 9763, *Z. rouxi* ATCC 28253.

The cylinder-plate method was used in the preliminary antimicrobial activity tests [18]. A suspension of the tested compound (20 mg/cm^3^, 0.05 cm^3^, in 0.08 M phosphate buffer, pH 7.0, containing 10 % DMSO) was placed in the cylinder. The cylinders were placed on a Muller–Hinton 2 or Sabouraud agar plate inoculated with one of the tested strains. The bacterial strains were incubated at 37 °C for 24 h and the fungal strains at 30 °C for 48 h. Minimal inhibitory concentration (MIC) was obtained by mixing with 19 cm^3^ Mueller–Hinton 2 agar and cooling to 56 °C with 1 cm^3^ of the appropriate dilution of the tested compound. Then, 2 × 10^−3^ cm^3^ of a particular cell suspension of optical density 0.5 unit on the McFarland scale was applied to the surface of the agar. The lowest concentration of tested compound which totally inhibited growth of the examined strain was evaluated as MIC value [19]. For control samples, MIC values of ciprofloxacin ranged between 0.14 and 0.37 × 10^−3^ μmol/cm^3^ for bacterial strains and MIC values of fluconazole ranged between 3.9 × 10^−4^ and 8.4 × 10^−1^ μmol/cm^3^ for yeast strains.

### Crystallography

Crystals of **1c** suitable for X-ray analysis were grown by slow evaporation of a solution in toluene–isopropanol (1:1). Diffraction data were collected on an Oxford Diffraction SuperNova diffractometer using CuK_α_ radiation at room temperature. Data reduction was performed with SuperNova software [20]. The unit cell parameters were determined by least-squares treatment of setting angles of the highest-intensity reflections chosen from the whole experiment. The structure was solved by direct methods, by use of SHELXS-97 software, and refined on *F* ^2^ by the full-matrix least-squares method, again by use of SHELXL97 software [21]. Two reflections were excluded from the reflection file because of their large $(\left| {F_{o} } \right|^{ 2} - \left| {F_{c} } \right|^{ 2} )$ differences. The function $\Upsigma w\left( {\left| {F_{\text{o}} } \right|^{ 2} - \left| {F_{\text{c}} } \right|^{ 2} } \right)^{ 2}$ was minimized with $w^{ - 1} = [\sigma^{ 2} \left( {F_{\text{o}} } \right)^{ 2} + \left( {0. 1 2 3 4P} \right)^{ 2} + 0. 3 5 6 8P]$, where $P = \left( {F_{\text{o}}^{ 2} + 2F_{\text{c}}^{ 2} } \right)/ 3$.

Non-hydrogen atoms were refined with anisotropic thermal data and the atoms of *O*-aminoethyl substituent were found to be disordered. So, the C12, C13A, and C13B atoms were located in two alternative positions and their occupancies were refined to 0.487(5) for C12A/C13A/C13B and 0.513(5) for C12B/C13C/C13D. The coordinates of the hydrogen atoms were generated geometrically and refined “riding” on their parent atoms with *U*_iso_ set at 1.2 (1.5 for methyl group) times *U*_eq_ of the appropriate carrier atom. All details concerning data collection, crystal data, and structure refinement are given in Table 5. The supplementary information in the CIF form is available from Cambridge Crystallographic Database Centre, no. CCDC-949328.

**Table 5:** Crystal data, data collection, and structure refinement for **1c**

Compound	**1c**
Empirical formula	C_18_H_23_NO_5_
Formula weight	333.37
*T*/K	293(2)
Wavelength/Å	1.54178
Crystal system, space group	Monoclinic, *P* 2(1)/*n*
Unit cell dimensions	
*a*/Å	8.1681(1)
*b*/Å	7.4276(1)
*c*/Å	27.3017(3)
*β*/°	94.218(1)
Volume/Å^3^	1,651.89(4)
*Z*, *D*_*x*_/mg m^−3^	4, 1.340
*μ*/mm^−1^	0.805
*F*(000)	712
*θ* range for data collection/°	5.55–88.24
*hkl* range	−10 ≤ *h* ≤ 10
	−9 ≤ *k* ≤ 7
	−33 ≤ *l* ≤ 33
Reflections	
Collected	17,343
Unique (*R*_int_)	3,523 (0.021)
Observed (*I* > 2*σ(I)*)	3,324
Data/restraints/parameters	3,523/0/236
Goodness-of-fit on *F* ^2^	1.007
*R*(*F*) [*I* > 2*σ*(*I*)]	0.0629
*wR*(*F* ^2^) (all data)	0.1921
Max/min. ∆*ρ*e/Å^−3^	0.323/–0.278
